# The Westward Journey of Alfalfa Leaf Curl Virus

**DOI:** 10.3390/v10100542

**Published:** 2018-10-04

**Authors:** Zohreh Davoodi, Nicolás Bejerman, Cécile Richet, Denis Filloux, Safaa G. Kumari, Elisavet K. Chatzivassiliou, Serge Galzi, Charlotte Julian, Samira Samarfard, Verónica Trucco, Fabián Giolitti, Elvira Fiallo-Olivé, Jesús Navas-Castillo, Nader Asaad, Abdul Rahman Moukahel, Jomana Hijazi, Samia Mghandef, Jahangir Heydarnejad, Hossein Massumi, Arvind Varsani, Ralf G. Dietzgen, Gordon W. Harkins, Darren P. Martin, Philippe Roumagnac

**Affiliations:** 1 Department of Plant Protection, College of Agriculture, Shahid Bahonar University of Kerman, Kerman 761, Iran; z.davoodi@agr.uk.ac.ir (Z.D.); jheydarnejad@yahoo.com (J.H.); masoomi@uk.ac.ir (H.M.); 2 Consejo Nacional de Investigaciones Científicas y Técnicas (CONICET), Godoy Cruz 2290, CABA, Argentina; nicobejerman@gmail.com; 3 Instituto de Patología Vegetal–Centro de Investigaciones Agropecuarias–Instituto Nacional de Tecnología Agropecuaria (IPAVE-CIAP-INTA), 5020 Córdoba, Argentina; trucco.veronica@inta.gob.ar (V.T.); giolitti.fabian@inta.gob.ar (F.G.); 4 French Agricultural Research Centre for International Development (CIRAD), BGPI, Montpellier 34398, France; cecile.richet@supagro.fr (C.R.); denis.filloux@cirad.fr (D.F.); serge.galzi@cirad.fr (S.G.); charlotte.julian@cirad.fr (C.J.); 5 BGPI, INRA, CIRAD, SupAgro, Univ Montpellier, Montpellier 34398, France; 6 International Center for Agricultural Research in the Dry Areas (ICARDA), Beqa’a Valley, Zahle 1801, Lebanon; s.kumari@cgiar.org (S.G.K.); a.moukahel@cgiar.org (A.R.M.); 7 Plant Pathology Laboratory, Department of Crop Science, Agricultural University of Athens, Athens 118 55, Votanikos, Greece; echatz@aua.gr; 8 Queensland Alliance for Agriculture and Food Innovation, The University of Queensland, St Lucia 4072, Queensland, Australia; s.samarfard@uq.edu.au (S.S.); r.dietzgen@uq.edu.au (R.G.D.); 9 Instituto de Hortofruticultura Subtropical y Mediterránea “La Mayora”, Consejo Superior de Investigaciones Científicas-Universidad de Málaga (IHSM-CSIC-UMA), Avenida Dr. Wienberg s/n, 29750 Algarrobo-Costa, Málaga 29001, Spain; efiallo@eelm.csic.es (E.F.-O.); jnavas@eelm.csic.es (J.N.-C.); 10 General Commission for Scientific Agricultural Research (GCSAR), 561-0817 Al-Ghab, Hama, Syria; asaad_nader@yahoo.com; 11 National Center for Agricultural Research and Extension (NCARE), 19381 Al-Baqah, Jordan; hijazijomana@yahoo.com; 12 Virology Laboratory, ICARDA, Ariana 2049, Tunisia; mghandefsamia91@gmail.com; 13 The Biodesign Center for Fundamental and Applied Microbiomics, Center for Evolution and Medicine and School of Life sciences, Arizona State University, Tempe, AZ 85287-5001, USA; arvind.varsani@asu.edu; 14 Structural Biology Research Unit, Department of Clinical Laboratory Sciences, University of Cape Town, Cape Town 7925, Western Cape, South Africa; 15 South African MRC Bioinformatics Unit, South African National Bioinformatics Institute, University of the Western Cape, Bellville 7535, Western Cape, South Africa; gordon@sanbi.ac.za; 16 Computational Biology Group, Institute of Infectious Diseases and Molecular Medicine, University of Cape Town, Cape Town 7925, Western Cape, South Africa; darrenpatrickmartin@gmail.com

**Keywords:** Alfalfa leaf curl virus, geminivirus, alfalfa, evolutionary history

## Abstract

Alfalfa leaf curl virus (ALCV), which causes severe disease symptoms in alfalfa (*Medicago sativa* L.) and is transmitted by the widespread aphid species, *Aphis craccivora* Koch, has been found throughout the Mediterranean basin as well as in Iran and Argentina. Here we reconstruct the evolutionary history of ALCV and attempt to determine whether the recent discovery and widespread detection of ALCV is attributable either to past diagnostic biases or to the emergence and global spread of the virus over the past few years. One hundred and twenty ALCV complete genome sequences recovered from ten countries were analyzed and four ALCV genotypes (ALCV-A, ALCV-B, ALCV-C, and ALCV-D) were clearly distinguished. We further confirm that ALCV isolates are highly recombinogenic and that recombination has been a major determinant in the origins of the various genotypes. Collectively, the sequence data support the hypothesis that, of all the analyzed locations, ALCV likely emerged and diversified in the Middle East before spreading to the western Mediterranean basin and Argentina.

## 1. Introduction

Although geminiviruses have been intensively studied since the 1970s [1,2], it is only since the recent development and application of viral metagenomics-based approaches that the true diversity of this group of viruses is starting to become apparent [3,4]. The genus *Capulavirus* is just one of several new genera in the family *Geminiviridae* that have been established to accommodate some of the novel and diverse geminiviruses that have been discovered over the past ten years [5]. The genus *Capulavirus* currently contains four species (*Alfalfa leaf curl virus*, *Euphorbia caput-medusae latent virus*, *French bean severe leaf curl virus*, and *Plantago lanceolata latent virus*) that infect both cultivated and non-cultivated plants in southern and northern Europe, the Indian subcontinent, and South Africa [6,7,8].

Alfalfa leaf curl virus (ALCV), which causes severe disease symptoms in alfalfa (*Medicago sativa* L.), is transmitted by the widespread aphid species, *Aphis craccivora* Koch [7,9]. A study of ALCV isolates collected between 2010 and 2014 in France and Spain [10,11] revealed that both intra- and inter-species recombination has played a significant role in the evolution of ALCV. In addition, this study suggested that ALCV was probably widely distributed across the Mediterranean basin [11]. Consistent with this hypothesis, ALCV was subsequently reported in 2018 from Jordan, Syria, Lebanon, and Tunisia [12] as well as from the non-Mediterranean countries Iran [13] and Argentina [14].

The discovery of ALCV in Argentina and the Mediterranean basin so soon after its initial characterization raises questions regarding the potential global emergence of this virus: does its discovery in these far-flung regions imply that a “true” global and potentially damaging geminivirus emergence event has recently occurred, or, more prosaically, does it merely reflect the fact that broader and more intensive sampling of plant material coupled with more sensitive virus detection techniques have only recently revealed an epidemic that has been ongoing for tens (or even hundreds) of years? Interestingly, alfalfa symptoms resembling those caused by ALCV, such as plant stunting and leaf curling, crumpling, and shriveling, have been reported since the 1950s in Europe (including France, Bulgaria, Romania, and Spain) and the Middle East (Saudi Arabia) [15,16,17,18,19]. The etiological agent of this alfalfa disease, which was shown to be transmissible by both grafting and *A. craccivora* [18,20], was identified as a rhabdovirus, referred to as lucerne enation virus (LEV) [15]. However, further studies revealed that different types of symptoms were observed depending on the mode of transmission [19], suggesting that in several instances where alfalfa diseases have been attributed to LEV, one or more additional viruses may have been co-infecting plants together with LEV. If this “Trojan horse” hypothesis was true, the apparently sudden occurrence of ALCV could simply reflect diagnostic biases rather than the recent emergence and global spread of the virus. Addressing this question is of great importance since alfalfa is the most-cultivated perennial forage legume in temperate regions of the world [21].

Here we examine 120 ALCV full genome sequences recovered from ten countries to reveal four distinct ALCV genotypes (ALCV-A, ALCV-B, ALCV-C, and ALCV-D) with the isolates of each genotype sharing <93% genome-wide pairwise identity with those of the other three genotypes. In addition, we confirm that ALCV isolates are highly recombinogenic and that recombination has been a major determinant in their origins. Phylogenetic analyses also suggest that, of all the countries analyzed, ALCV most likely originated in Iran and diversified in the Middle East before spreading to the Mediterranean basin and then onwards to Argentina.

## 2. Material and Methods

### 2.1. Plant Sampling

Leaves from 564 alfalfa plants that were either asymptomatic or presented with conspicuous disease symptoms (including plant stunting or varying degrees of leaf curling, crumpling, and shriveling), were collected from 2010 to 2017 in Argentina (41 plant samples from 17 regions), France (73 samples, 5 regions), Greece (36 samples, 8 regions), Iran (141 samples, 10 regions), Italy (50 samples, 6 regions), Jordan (11 samples, 2 regions), Lebanon (5 samples), Namibia (25 samples, 1 region), South Africa (91 samples, 3 regions), Spain (62 samples, 3 regions), Syria (12 samples, 1 region), and Tunisia (17 samples, 2 regions).

### 2.2. DNA Extraction and PCR-Mediated Alfalfa Leaf Curl Virus Detection

Total DNA from the French, Greek, Italian, Jordanian, Lebanese, Namibian, South African, Spanish, Syrian, and Tunisian alfalfa samples was extracted using the DNeasy Plant Mini Kit (Qiagen, Hilden, Germany) following the manufacturer’s protocol. Total DNA from alfalfa samples from Argentina and Iran was extracted using the cetyltrimethylammonium bromide (CTAB) method [22]. PCR-based detection of ALCV from alfalfa plants collected in all countries other than Argentina and Iran was performed using the primer pair ALCV-187F (5′-TGG AAT ATT GTG CTG CTT GG-3′) and ALCV-971R (5′-ATT TTG GGA CTT GTG CTC CA-3′), as previously described in Bernardo et al. (2016) [11]. The presence of ALCV from the Iranian alfalfa samples was tested using PCR with the primer pair Gemini F1 (5′-ATG ATG GAT AAT TCA AAC CC-3′) and Gemini R2 (5′-CAC CTC CAC TGT CTT GTC CA-3′), as described in Davoodi et al. (2018) [13]. The presence of ALCV from the Argentinean alfalfa samples was tested using PCR with the KAPA HiFi HotStart PCR Kit (Kapa Biosystems, Wilmington, MA, USA) following the manufacturer’s protocol with the primer pair ALCV CPF (5′- GAG AAC GTA TGG ATT GGT C-3′) and ALCV CPR (5′-AGT GTA TGC GTT CTT CTG G-3′). Amplification conditions consisted of: 95 °C for 3 min, 35 cycles at 98 °C for 20 s, 58 °C for 15 s, 72 °C for 45 s, and a final extension at 72 °C for 1 min.

### 2.3. Cloning and Sequencing of ALCV Full Length Genomes

Fifty-one out of the 170 alfalfa samples that tested positive for ALCV included plants sampled in France (*n* = 3 out of 27 samples that tested positive for ALCV), Greece (*n* = 3/7), Iran (*n* = 14/20), Italy (*n* = 7/9), Jordan (*n* = 3/11), Lebanon (*n* = 2/5), Spain (*n* = 11/27), Syria (*n* = 3/12), and Tunisia (*n* = 5/17). DNA extracted from these 51 samples was used as a template for PCR amplification of complete ALCV genomes using the HotStarTaq Plus Master Mix Kit (Qiagen, Hilden, Germany) following the manufacturer’s protocol and the pair of abutting primers, Cap-ncoIF (5′-CCA TGG CCT TCA AAG GTA GCC CAA TTC AAY ATG G-3′) and Cap-ncoIR (5′-CCA TGG GGC CTT ATY CCT CKG YGA TCG-3′), which contain an overlapping *Nco*I site as described in Bernardo et al. (2016) [11]. Amplification conditions were 95 °C for 5 min, 35 cycles of 94 °C for 20 s, 60 °C for 30 s, 68 °C for 165 s, and finally 72 °C for 180 s. The amplicons were gel purified using the PCR Clean-Up System (Promega, Madison, WI, USA), cloned into pGEM-T Easy (Promega, Madison, WI, USA) and Sanger sequenced using primer walking at Genewiz (South Plainfield, NJ, USA). DNA of 34 samples that tested positive for ALCV from Argentina were used as a template for PCR amplification of the complete genome using the KAPA HiFi HotStart PCR Kit (Kapa Biosystems, Wilmington, MA, USA) following the manufacturer’s protocol and the pair of abutting primers CG-F (5´-CTC AAT GAA TCC ACA TCC AAG-3´) and CG-R (5´-CGA GGA ACT CGG ACT TGG A-3´). Amplification conditions were 95 °C for 3 min, 35 cycles of 98 °C for 20 s, 57 °C for 15 s, 72 °C for 165 s, and finally 72 °C for 180 s. The amplicons were gel purified using the PCR Clean-Up System, cloned in pGEM-T Easy and Sanger sequenced using primer walking at Macrogen Inc. (Seoul, South Korea).

### 2.4. Pairwise-Distance, Phylogenetic, and Recombination Analyses

Genome-wide pairwise comparisons of 85 newly determined ALCV genome sequences together with 35 previously determined ALCV genome sequences were done using the Sequence Demarcation Tool SDT v1.2 [23]. The 120 ALCV sequences were aligned together with the capulavirus Euphorbia caput-medusae latent virus (EcmLV, GenBank HF921459 [6]; chosen as outgroup) using MUSCLE [24] with default settings. Evidence of potential recombination events was detected within the 120 ALCV full-genome alignment using the RDP, GENECONV, BOOTSCAN, MAXIMUM CHI SQUARE, CHIMAERA, SISCAN and 3SEQ recombination detection methods that are implemented in RDP4.94 (using default settings [25]). Only recombination events that were detected with two or more detection methods, and had significant phylogenetic support, were considered credible evidence of recombination.

A maximum likelihood (ML) phylogenetic tree of the 120 aligned full genome sequences, with recombinant regions removed, was constructed using PhyML3 [26] implemented in MEGA 7.0.26 [27] with JC + G selected as the best fit nucleotide substitution model and 1000 non-parametric bootstrap replicates. The tree was rooted with EcmLV. Branches with less than 30% bootstrap support were collapsed using TreeGraph2 [28]. In addition, a maximum likelihood (ML) phylogenetic tree of the 56 aligned ALCV-A full genome sequences, with recombinant regions removed, was constructed using PhyML3 [26] implemented in MEGA 7.0.26 [27] with T92 + I + G selected as the best fit nucleotide substitution model and 1000 non-parametric bootstrap replicates. The tree was rooted with one Iranian isolate from ALCV-C (GenBank accession number: MH085199). Branches with less than 50% bootstrap support were collapsed using TreeGraph2 [28].

The evolutionary relationships of ALCV isolates were reconstructed using replication-associated protein (Rep) and coat protein (CP) amino acid sequences. Datasets consisting of 120 predicted ALCV Rep and CP amino acid sequences together with the corresponding homologous sequence from EcmLV, chosen as a capulavirus outgroup, was used to root the Rep and CP phylogenies. Predicted Rep and CP amino acid sequences were aligned using MUSCLE [24] with default settings. Maximum likelihood phylogenetic trees of the Rep and CP were inferred using PhyML3 [26] implemented in MEGA with the Jones–Taylor–Thornton (JTT) + G amino acid substitution model chosen as the best-fit using ProtTest [29]. Five hundred bootstrap replicates were used to test the support of branches. Branches with less than 50% bootstrap support were collapsed using TreeGraph2.

### 2.5. Statistical Analyses

Correlation of geographic and genetic distance were assessed using a Mantel test implemented in GenAlEx [30] (with 999 random permutations used to test the significance of the correlation). Whereas the genetic distance matrix was obtained for the 56 aligned ALCV-A full genome sequences, with recombinant regions removed using MEGA 7.0.26 (CLUSTALW alignment followed by uncorrected pairwise distance estimation with pairwise deletion of gaps), the geographic distance matrix was obtained using the program Geographic Distance Matrix Simulator 1.2.3 (http://biodiversityinformatics.amnh.org/open_source/gdmg).

## 3. Results and Discussion

### 3.1. Genetic Diversity of ALCV Isolates

One hundred and seventy alfalfa samples tested positive for ALCV, including plants sampled from Argentina (35/41), France (27/73), Greece (7/36), Iran (20/141), Italy (9/50), Jordan (11/11), Lebanon (5/5), Spain (27/62), Syria (12/12), and Tunisia (17/17). ALCV was only ever detected from symptomatic plants but never from asymptomatic plants. All samples from Namibia and South Africa tested negative for ALCV. Overall, 85 full length genome sequences were reported in this study (GenBank accession numbers: MG792020–MG792053; MH603810–MH603860), which were analyzed together with the 35 complete genome sequences recovered in previous studies [7,11,12,13,14] (Supplementary Materials Table S1). The sizes of these 120 complete ALCV genome sequences ranged from 2712 nt to 2769 nt, and they all shared >80.2% genome-wide pairwise identity. This degree of sequence identity is above the species demarcation threshold (78%) recommended for members of the *Capulavirus* genus [5], and all 120 isolates were therefore considered to belong to the species *Alfalfa leaf curl virus*. In addition, pairwise identity distribution analysis of the 120 ALCV complete genome sequences identified two clear troughs at 87% and 93% (Supplementary Figure S1) that might indicate credible genotype demarcation thresholds. Since implementing a cutoff for ALCV genotype demarcation at 93% yielded the lowest number of genotype-level classification conflicts, we chose 93% as a tentative genotype demarcation threshold for ALCV.

**Figure 1:**
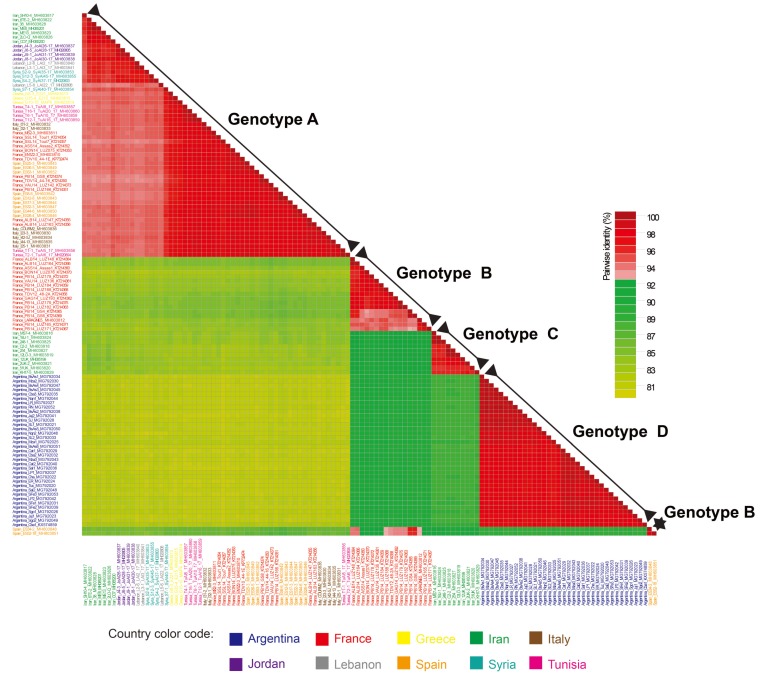
A “two-color” genome-wide pairwise identity matrix inferred using SDTv1.2 [23] showing that a tentative ALCV genotype demarcation threshold of 93% allows discrimination of the four ALCV genotypes (ALCV-A, ALCV-B, ALCV-C, and ALCV-D). Percent sequence identity is indicated by the color-coded boxes.

Using this threshold level, four ALCV genotypes (ALCV-A, ALCV-B, ALCV-C, and ALCV-D) were clearly distinguished (Figure 1). ALCV-A isolates (*n* = 56) were present in almost all of the countries where ALCV has so far been reported, including France, Greece, Iran, Italy, Jordan, Lebanon, Spain, Syria, and Tunisia. By contrast, ALCV-B (*n* = 19) was only recovered in France and Spain and ALCV-C (*n* = 10) and ALCV-D (*n* = 35) have to date, only been found in Iran and Argentina, respectively (Figure 1). Averages of genome-wide pairwise identities between isolates of each genotype indicate that ALCV-A and ALCV-B were the most genetically diverse groups (95.7% and 96.4%, respectively) with ALCV-C isolates sharing, on average, 97.1% identity and ALCV-D isolates sharing 98.1% (Table 1). Specifically, two outlier isolates from Spain (ES34-2 and ES52-18), with strong evidence of recombination, were assigned to genotype ALCV-B because they share >93% genome-wide pairwise identity with 10 out of the 17 other isolates of genotype ALCV-B (Figure 1). While ALCV-A and ALCV-D isolates all contained at least seven recognizable open reading frames (ORFs) with more than 30 aa, including four virion-sense ORFs (V1, V2, V3, and V4) and three complementary-sense ORFs (C1, C2, and C3), ALCV-B and ALCV-C isolates lacked the V2 ORF. Both of these genomic organizations have been previously described [11].

### 3.2. Phylogenetic and Recombination Analyses

Twenty-one unique recombination events were detected (Table 2). Notably, all of the examined ALCV isolates displayed traces of recombination events with, on average, two events being evident within each analyzed isolate. Ten out of the 21 ALCV recombination events apparently involved intra-species transfers of sequences between ALCV variants whereas the other 11 detected events apparently involved inter-species sequence transfers (Table 2).

**Figure 2:**
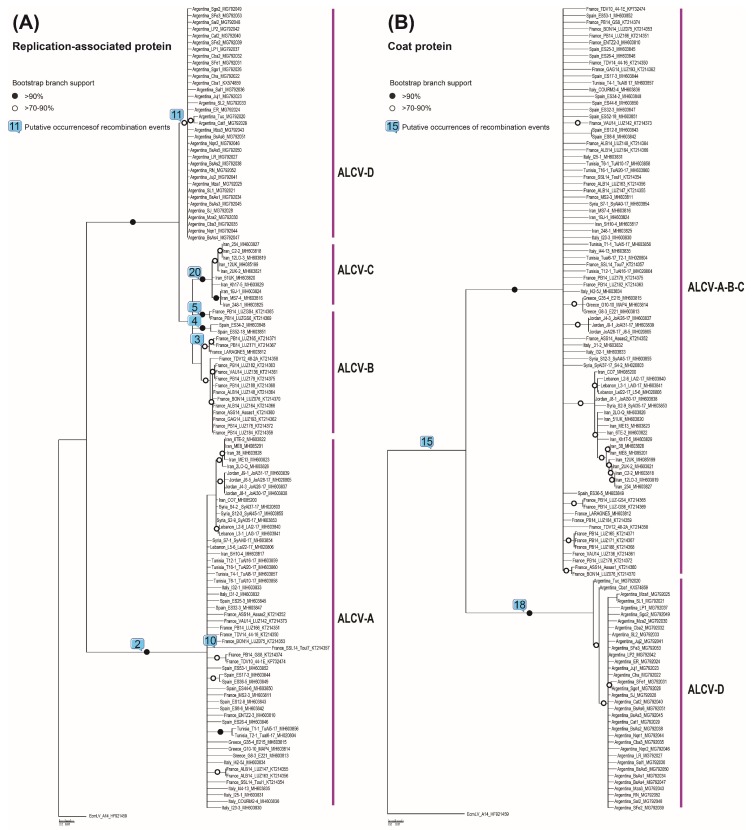
Maximum-likelihood phylogenetic trees of predicted replication-associated protein (Rep) (**A**) and coat protein (CP) (**B**) amino acid sequences of 120 ALCV isolates, both rooted with Euphorbia caput-medusae latent virus CP and Rep amino acid sequences. Branches with less than 50% bootstrap support were collapsed. Branches associated with a black dot have bootstrap supports above 90% whereas those with white dots have bootstrap supports above 70%. Putative occurrences of recombination events (numbers within squares correspond to the number of the event as listed in Table 2) are depicted on branches of both phylogenetic trees.

Interestingly, event 2 (corresponding with event 1 in Bernardo et al. (2016) [11]), which involved an inter-species sequence transfer of the entire replication-associated protein (*rep*) gene, accounted for the clear divergence of the ALCV-A isolates from all of the other ALCV genotypes (Figure 2A). At least five recombination events involving the partial replacement of the *rep* gene (events 3, 4, 5, 11, and 20) also appear to be the primary causes of the genetic differences found between ALCV-B, -C, and -D isolates (Figure 2A).

The coat protein (*cp*) genes of the ALCV isolates have apparently been less affected by inter-species sequence exchanges than the *rep* genes of these isolates (Table 2); a factor that was likely responsible for the ALCV CP proteins displaying less variability than the ALCV REP proteins. The ALCV CP protein is likely to play a central role in insect transmission and virus genome packaging [31] and, as appears to be the case for begomoviruses [32], the *cp* gene is either less prone to recombination than the *rep* gene, or is more prone to recombination-induced functional impairments than the *rep* gene such that viruses with recombinant *cp* genes are selected against. However, event 15 (corresponding with event 9 in Bernardo et al. (2016) [11]), which is detectable in all analyzed ALCV isolates other than those in the ALCV-D genotype (all from Argentina) and eleven of the ALCV-B isolates from France and Spain, involved the transfer of a fragment of the *cp*. If this recombination event predated the most recent common ancestor of the analyzed ALCV isolates, then, in the ALCV-D isolates and the eleven ALCV-B isolates, evidence of this event may have been obscured by subsequent recombination events 6, 7, 13, 14, and 17 (Table 2).

The ALCV-D genotype has also probably arisen following two major recombination events collectively involving transfers of approximately 2/3 of the genome: one involving acquisition of a *rep* gene (event 11, Figure 2A) and the other involving acquisition of the *cp* gene (event 18, Figure 2B).

These results indicate that, as is the case with other geminiviruses, ALCV (and probably the other capulaviruses) are likely highly recombinogenic and, as a consequence, have the potential for rapid adaptive evolution [33,34,35,36]. Given that ALCV is transmitted by *A. craccivora*, which feeds on a diverse range of host plant species [37], the host range encounters that ALCV might naturally have with other geminiviruses is probably high. This alone might account for 38% of the recombination events that were identified here as involving unknown geminiviruses. In addition, while *A. craccivora* has been reported on all continents except the Antarctic [38], and displays some morphological and genetic evidence of differentiation into host races [39], one can expect that geographical populations of this aphid could have driven the microevolution of the viruses that they carry and, by extension, promoted the rapid adaptive evolution of these viruses by recombination: as is suggested by the diversity of the Argentinean ALCV isolates.

### 3.3. Geographic Distribution of ALCV

**Figure 3:**
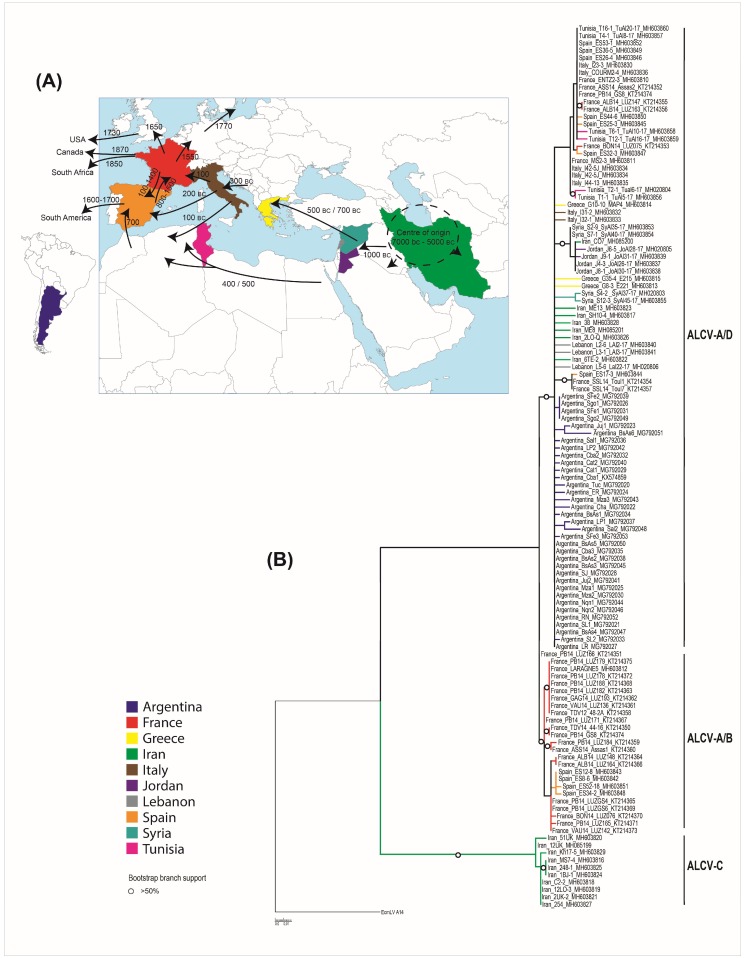
(**A**) Map of Europe, the Middle East, and South America indicating the different routes and approximate dates of spread of cultivated alfalfa from its center of origin (this map is adapted from Prosperi et al. (2014) [44]). Countries from which ALCV has been reported are highlighted in several colors corresponding to those used in the phylogenetic tree. (**B**) A maximum likelihood phylogenetic tree of the 120 aligned ALCV recombination-free genome sequences, with JC + G selected as the best fit nucleotide substitution model and 1000 non-parametric bootstrap replicates. The tree was rooted with Euphorbia caput-medusae latent virus. Branches with less than 30% bootstrap support were collapsed. Branches associated with a dotted circle have bootstrap support values above 50%.

While ALCV was initially reported from southern France and northern Spain, we show here that ALCV is found in diverse geographical and climatic zones of both the Old and New Worlds (Figure 3A), including temperate oceanic (Rodez/France or Buenos Aires/Argentina), continental (Entzeim/France), mountainous (Courmayeur/Italy), cold semi-desert (Isfahan/Iran), hot semi-desert (Kerman/Iran), or subtropical (Jujuy/Argentina) climatic zones. Given the worldwide distribution of *A. craccivora* and the “climatic flexibility” of ALCV, it is plausible that few natural barriers exist that would effectively limit the global spread of this virus [38]. Even the presently known distribution of ALCV suggests that it is capable of infecting a range of alfalfa varieties within the *M. sativa* species complex, including *M. sativa* subsp. *sativa* and probably *M. sativa* subsp. *falcata*.

Although the natural host range of ALCV is unknown, it is plausible that even without alternative hosts, ALCV could persist in “wild” alfalfa populations that are commonly found within a variety of unmanaged habitats. If ALCV has a broad host range, as is commonly the case with geminiviruses, then it will be very difficult to control the local spread of the virus wherever it is introduced to in the world. Identification of alfalfa cultivars that are tolerant or resistant to *A. craccivora* could constitute a sustainable strategy to moderate the impacts of ALCV wherever it occurs.

It must be emphasized that the currently known geographical range of ALCV excludes some of the regions that were investigated in this study. While the virus was detected in every country within the Mediterranean basin and the Middle East from which samples were obtained, it was not detected in samples from South Africa and Namibia, where alfalfa has been grown since the mid 1800 s (Figure 3A).

By contrast, we found that ALCV was widespread in Argentina, occurring in all 17 alfalfa-growing regions of the country from where samples were obtained. The 35 genome sequences from Argentinean samples collected between 2010 and 2017 were genetically highly homogeneous, sharing an average of 98.1% genome-wide pairwise identity. While this degree of diversity is consistent with the hypothesis that the Argentinean ALCV population was founded by a single introduced ALCV variant, our recombination analysis indicates that the descendants of this founder virus have undergone two significant inter-species recombination events that replaced 2/3 of the original genome, including the entire *rep* and *cp* genes with sequences from either a distantly related ALCV genotype or from a different *Capulavirus* species. One of the Argentinean isolates harbors traces of an additional minor recombination event (event 21, Table 2) that occurred in the large intergenic region. Collectively, the widespread distribution of ALCV in Argentina, the low diversity of the Argentinean ALCV population, and the fact that all of the isolates appear to have descended from the same recombinant ancestor, suggest that the virus was probably introduced only once and has subsequently spread throughout the country. This may have involved the efficient, large scale, and long-range transmission of the virus by *A. craccivora*. This is plausible since studies focusing on another persistently *A. craccivora* transmitted circular ssDNA virus (subterranean clover stunt virus, SCSV, *Nanoviridae*) have revealed that *A. craccivora* in Australia can migrate over several hundred kilometers from the coastal areas to cause SCSV re-infestation of pastures in the arid regions of southeast Australia [40]. Another possibility is that the same ALCV variant may have been introduced throughout Argentina in infected planting material such as seeds. Although seed-transmission has never been demonstrated for capulaviruses, this scenario cannot be completely ruled out as several recent studies have confirmed that geminiviruses belonging to at least three genera (*Begomovirus*, *Becurtovirus*, and *Curtovirus*) can be seed-transmitted [41,42,43].

### 3.4. Geographical Origin of ALCV

The ML phylogenetic tree of all 120 aligned ALCV complete genome sequences (with recombinationally-derived genome fragments removed and rooted with EcmLV) indicated that, among the locations from which samples were analyzed, the most recent common ancestor (MRCA) was probably located in Iran (Figure 3B). The three main ALCV lineages diverged from this MRCA, to subsequently form the ALCV-C, ALCV-B, ALCV-A, and ALCV-D genotypes (Figure 3B). The genotype A/D lineage probably experienced three major recombination events: Event 2 that involved the acquisition of a *rep* gene sequence from an unknown capulavirus and yielded genotype ALCV-A, and Events 11 and 18 that respectively involved the transfers of *rep* and *cp* genes from a divergent currently undiscovered ALCV lineage or a currently undiscovered *Capulavirus* species, which together yielded genotype ALCV-D. Two recombination events between ancestral ALCV-A and ALCV-B viruses (Events 1 and 3 in our analysis) yielded a sub-clade within the genotype A/B lineage that contains *rep* gene sequences that are today found in viruses that would otherwise be classified as belonging to the ALCV-A genotype (represented by eight isolates in our analysis).

**Figure 4:**
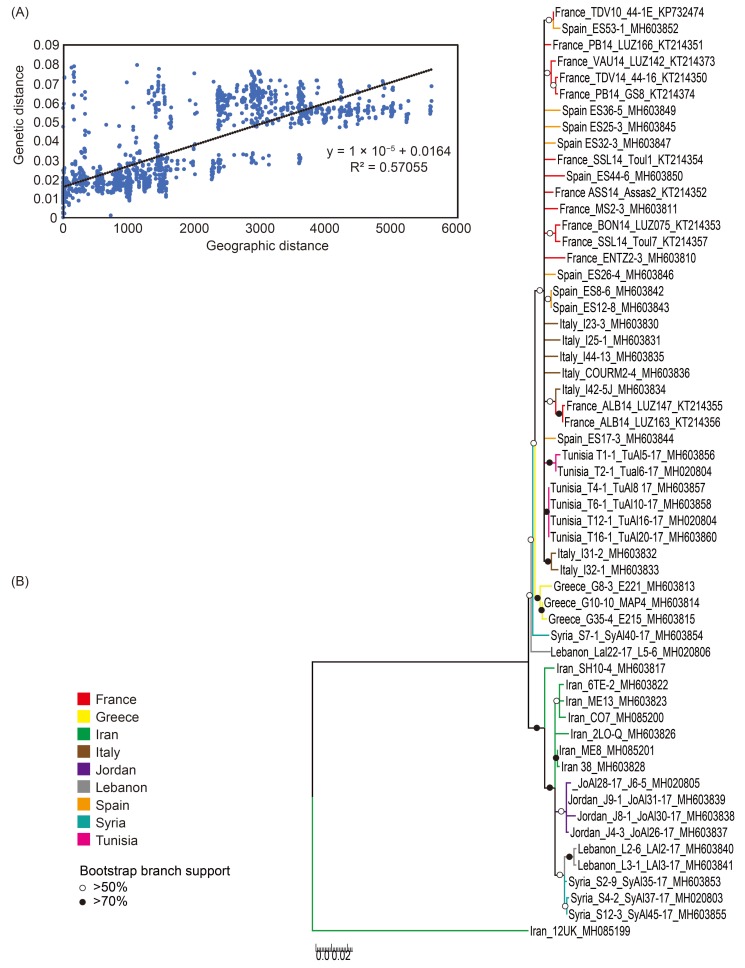
(**A**) Relationship of pairwise genetic distance of the 56 aligned ALCV-A full genome sequences with pairwise geographical distance (km). SSx (sum of products of x matrix elements) = 3.143 × 10^9^; SSy (sum of products of y matrix elements) = 0.657; SPxy (sum of cross products of corresponding elements of the x and y matrices) 3.433 × 10^4^; Rxy (Mantel correlation coefficient) = 0.755 and *P* (rxy-rand ≥ rxy-data); probability of Rxy based on 999 standard permutations across the full dataset = 0.001. (**B**) A maximum likelihood phylogenetic tree of the 56 aligned ALCV-A recombination-free genome sequences, with T92 + I + G selected as the best fit nucleotide substitution model and 1000 non-parametric bootstrap replicates. The tree was rooted with one Iranian isolate from ALCV-C (GenBank accession number: MH085199). Branches with less than 50% bootstrap support were collapsed. Branches associated with white and black dots have bootstrap support values above 50% and above 70%, respectively.

Interestingly, the averages of genome-wide pairwise identities of ALCV-A isolates from the Middle East (96.4%; isolates from Iran, Jordan, Lebanon, and Syria, Table 1) was lower than that of isolates from the western/central Mediterranean basin (98.0%; isolates from France, Italy, Greece Spain, and Tunisia, Table 1); this supports the hypothesis that genotype ALCV-A originated in the Middle East before spreading further westward. A Mantel test of association between total genetic distance and geographic distance revealed a strong correlation for the ALCV-A isolates (Rxy: correlation coefficient of Mantel test = 0.755; P (rxy-rand ≥ rxy-data: probability of positive autocorrelation (one tailed) = 0.001). This result implies a high degree of genetic isolation by distance within the Mediterranean and Middle East countries (Figure 4A).

In addition, the ML phylogenetic tree of all 56 aligned ALCV-A complete genome sequences (with recombinationally-derived genome fragments removed and rooted with an ALCV-C isolate), indicated that the ALCV-A MRCA was probably located in the Middle East (Figure 4B) and that the Greek isolates followed by the western Mediterranean isolates (from France, Italy, Spain, and Tunisia) became successively more divergent from the Middle Eastern isolates (Figure 4B). Collectively these results suggested that ALCV-A isolates originated in the Middle East and spread further westward.

Finally, the average genome-wide pairwise identity of the ALCV-B isolates, (96.4%; where all examined isolates have so far only been found in France and Spain), was lower than that of ALCV-C (97.1%), ALCV-D (98.1%), and ALCV-A from the western/central Mediterranean basin (98.0%); this supports the hypothesis that ALCV-B has been circulating for longer in Western Europe than the other ALCV genotypes. Collectively, the sequence data support the hypothesis that ALCV emerged and diversified in the Middle East (with Iran being the most probable origin of all the analyzed locations) before spreading possibly in at least two waves, to the Mediterranean basin and onwards from there to Argentina. This “two-waves” hypothesis fits well with the known domestication and spread history of alfalfa. While the first evidence of alfalfa cultivation can be traced to 7000 BC in Iran and/or central Asia [44], the plant was probably only domesticated in central Asia in approximately 5000 BC [45]. Alfalfa cultivation then spread to the Middle East around 1000 BC and from there to Greece by the Medes armies between 500 and 700 BC, and finally to Italy by 300 BC and the rest of the Roman Empire by 100 AD (Figure 3A). Alfalfa cultivation in Europe then declined during the Middle Ages but was later reintroduced there via Spain by the Arabs in approximately 700 AD (Figure 3A). Thereafter, alfalfa was introduced to South America by the Spanish in the sixteenth century (Figure 3A).

It is important to stress, however, that it remains to be determined whether the timescales of ALCV dissemination throughout the Mediterranean mirror those of alfalfa dissemination. Given that all the alfalfa samples examined here have been collected over the past eight years (2010–2017) there was insufficient temporal signal in our data sets to infer accurate and precise nucleotide substitution rates that would enable the estimation of the dates when ancestral sequences likely arrived in the countries from which they were sampled. Samples of ALCV-infected alfalfa plants collected in the early to mid-1900s might yield ALCV genome sequences that could provide the temporal signal that is necessary to infer whether ALCV did indeed disseminate together with the spread of alfalfa cultivation throughout the Mediterranean.

## Figures and Tables

**Table 1:** Averages of alfalfa leaf curl virus genome-wide pairwise identities.

Group of ALCV Isolates	Averages of Genome-Wide Pairwise Identities (%)
Genotype A isolates (*n* = 56)	95.7
Genotype B isolates (*n* = 19)	96.4
Genotype C isolates (*n* = 10)	97.1
Genotype D isolates (*n* = 35)	98.1
Genotype A isolates (Middle East, *n* = 18) ^a^	96.4
Genotype A isolates (Western Mediterranean basin, *n* = 38) ^b^	98.0

^a^ Genotype A isolates from the Middle East include isolates from Iran, Jordan, Lebanon, and Syria. ^b^ Genotype A isolates from the western Mediterranean basin include isolates from France, Italy, Greece, Spain, and Tunisia.

**Table 2:** Recombination events detected in alfalfa leaf curl virus isolates.

Event	Recombinant(s)	Major Parent	Minor Parent	Methods ^a^	Breakpoints Positions ^b^
1	France_VAU14_LUZ142_KT214373	France_PB14_GS4_KT214365	Unknown	GBMST	1420 (nad) ^c^–2736 (nad)
2	Jordan_J9-1_MH603839	EcmLV_A14_HF921459	Argentina_Cba1_KX574859	GBMCST	1407 (nad)–2704 (nad)
3	France_PB14_LUZ171_KT214367	Unknown	France_LARAGNE5_MH603812	RGBMCST	1332 (nad)–2193
4	Spain_ES34-2_MH603848	France_PB14_GS4_KT214365	Iran_SH10-4_MH603817	RGMCST	2097 (nad)–2196
5	France_PB14_GS4_KT214365	France_ALB14_LUZ148_KT214364	Spain_ES52-18_MH603851	RBMCT	1750 (nad)–2053
6	France_BON14_LUZ076_KT214370	France_GAG14_LUZ193_KT214362	Unknown	RGBMCST	434–1251
7	France_PB14_LUZ184_KT214359	France_TV12_48-2A_KT214358	France_LARAGNE5_MH603812	RGBMCST	845–1027
8	Iran_MS7-4_MH603816	Iran_12LO-3_MH603819	Iran_SH10-4_MH603817	RMCST	277–1295 (nad)
9	France_GAG14_LUZ193_KT214362	France_TDV12_48-2A_KT214358	Tunisia_T6-1_MH603858	MST	291–1261
10	France_SSL14_Toul7_KT214357	France_TDV10_44-1_KP732474	Unknown	RGMCST	2124–2355
11	Argentina_Sal2_MG792048	Unknown	Spain_ES52-18_MH603851	RBMCT	1259–2070 (nad)
12	Spain_ES52-18_MH603851	Unknown	France_PB14_LUZ179_KT214375	RGMCT	289 (nad)–1333
13	France_PB14_LUZ179_KT214375	France_LARAGNE5_MH603812	Tunisia_T2-1_MH020804	RGMCST	868–1333 (nad)
14	France_ASS14_Assas1_KT214360	France_PB14_LUZ171_KT214367	Italy_COURM2-4_MH603836	RGMCST	901 (nad)–1029
15	France_TDV10_44-1_KP732474	France_LARAGNE5_MH603812	EcmLV_A14_HF921459	RM	872–1053 (nad)
16	France_PB14_LUZ171_KT214367	France_PB14_LUZ179_KT214375	Spain_ES34-2_MH603848	RGS	2434–2537
17	France_LARAGNE5_MH603812	France_VAU14_LUZ142_KT214373	France_BON14_LUZ076_KT214370	RMC	844 - 1026
18	Argentina_Mza3_ MG792043	Unknown	France_LARAGNE5_MH603812	MC	211–1261 (nad)
19	Syria_S2-9_MH603853	Iran_38_MH603828	Spain_ES8-6_MH603842	MC	1797 (nad)–2093
20	Iran_2UK-2_MH603821	France_PB14_LUZ184_KT214359	EcmLV_A14_HF921459	RG	1976 (nad)–2016
21	Argentina_Nqn2_MG792046	Argentina_BsAs1_ MG792034	Unknown	RBT	2493 (nad)–2565

^a^ RDP (R), GENECONV (G), BOOTSCAN (B), MAXIMUM CHI SQUARE (M), CHIMAERA (C), SISCAN (S) and 3SEQ (T) recombination detection methods. ^b^ Begin and end breakpoints positions in the recombinant sequence. ^c^ nad: not accurately determined.

## Supplementary Materials

The following are available online at http://www.mdpi.com/1999-4915/10/10/542/s1, Table S1: Features of the 120 ALCV isolates analyzed in this study. Figure S1: Pairwise identity frequency distribution plot of 120 complete genome sequences of alfalfa leaf curl virus.
