# Supplementary material for: The Westward Journey of Alfalfa Leaf Curl Virus
Source: Viruses. 2018 Oct 4;10(10):542. doi: 10.3390/v10100542 (PMC6212810; doi:10.3390/v10100542)
Supplement: Supplementary file 1 [file viruses-10-00542-s001.pdf]

viruses

MDPI

Supplementary Material

Supplementary Figure 1

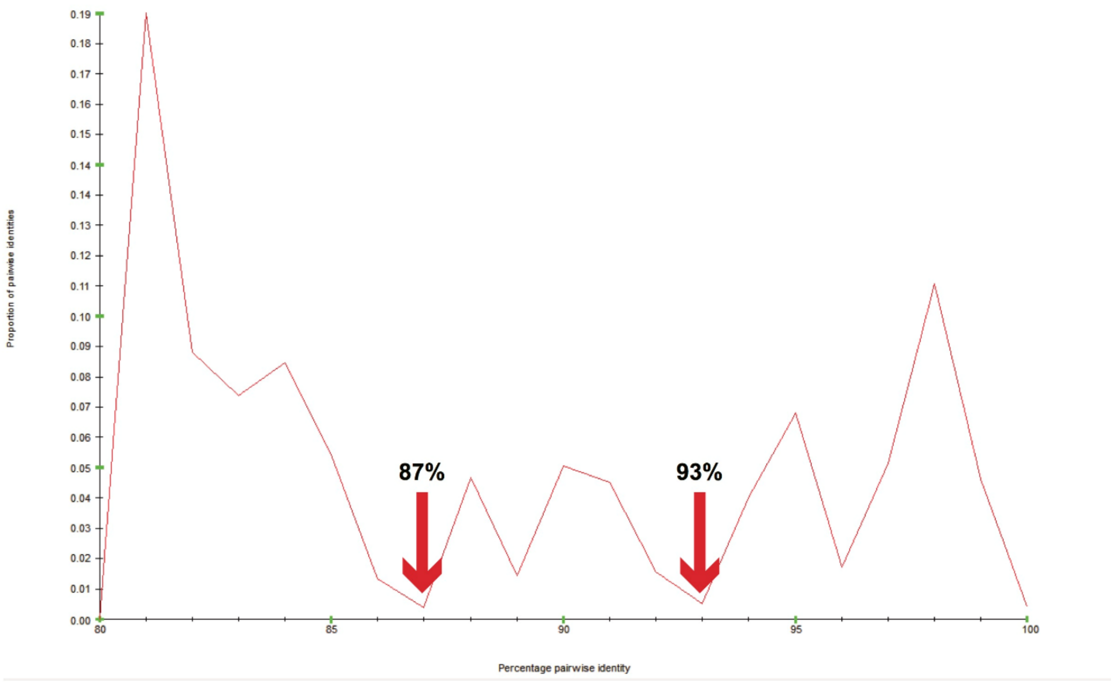

Figure S1. Percentage pairwise identities of 120 complete genome sequences of alfalfa leaf curl virus. Sequence demarcation thresholds at 87% and 93% are indicated with a red arrow

Table S1. Features of the 120 ALCV isolates analyzed in this study.

Isolate	Country	Region	Locality	GPS Coordinates	Year	Acc. number	Strain	Reference
BsAs1_Guamini	Argentina	Buenos Aires	Guaminí	36°53'54.60"S 62°23'31.20″W	2017	MG792034	D	This study
BsAs2_Saladillo	Argentina	Buenos Aires	Saladillo	35°40'19.56"S 59°48'44.64″W	2011	MG792038	D	This study
BsAs3_Pergamino	Argentina	Buenos Aires	Pergamino	33°54'58.32"S 60°34'18.12″W	2011	MG792045	D	This study
BsAs4_Hilario_Ascasubi	Argentina	Buenos Aires	Hilario Ascasubi	39°23'33.36"S 62°37'41.88″W	2015	MG792047	D	This study
BsAs5_Piedritas	Argentina	Buenos Aires	Piedritas	34°43'37.92"S 62°59'10.68″W	2016	MG792050	D	This study
BsAs6_Tandil	Argentina	Buenos Aires	Tandil	37°21'27.00"S 59°1'23.88″W	2017	MG792051	D	This study
Cat1_Capayan	Argentina	Catamarca	Capayán	28°46'28.56"S 66°2'52.80″W	2015	MG792029	D	This study
Cat2_Tinogasta	Argentina	Catamarca	Tinogasta	28°4'59.16"S 67°34'5.16″W	2015	MG792040	D	This study
Cba1_Manfredi	Argentina	Córdoba	Manfredi	31°50'44.52"S 63°44'51.00″W	2014	KX574859	D	Bejerman et al. 2018
Cba2_Calchin	Argentina	Córdoba	Calchín	31°39'55.08"S 63°11'6.72″W	2016	MG792032	D	This study
Cba3_Santa_Eufemia	Argentina	Córdoba	Santa Eufemia	33°12'24.84"S 63°17'6.36″W	2016	MG792035	D	This study
Cha_Charata	Argentina	Chaco	Charata	27°14'22.20"S 61°11'21.84″W	2012	MG792022	D	This study
ER_Victoria	Argentina	Entre Ríos	Victoria	32°34'26.76"S 60°3'52.56″W	2011	MG792024	D	This study
Juj1_Palma_Sola	Argentina	Jujuy	Palma Sola	23°58'10.20"S 64°17'53.52″W	2012	MG792023	D	This study
Juj2_Volcan	Argentina	Jujuy	Volcan	23°55'39.72"S 65°28'13.80″W	2012	MG792041	D	This study
LP1_Ojeda	Argentina	La Pampa	Ojeda	35°2'52.08"S 63°51'20.88″W	2010	MG792037	D	This study
LP2_General_Pico	Argentina	La Pampa	Gral Pico	35°42'5.76"S 63°45'54.00″W	2017	MG792042	D	This study
LR_Villa_Castelli	Argentina	La Rioja	Villa Castelli	29°1'23.16"S 68°13'0.48″W	2015	MG792027	D	This study
Mza1_Tulumaya	Argentina	Mendoza	Tulumaya	32°42'57.60"S 68°38'6.00″W	2012	MG792025	D	This study
Mza2_Malargue	Argentina	Mendoza	Malargue	35°29'42.72"S 69°35'9.96″W	2014	MG792030	D	This study
Mza3_Monte_Coman	Argentina	Mendoza	Monte Coman	34°38'22.20"S 67°59'51.36″W	2014	MG792043	D	This study
Nqn1_Chos_Malal	Argentina	Neuquén	Chos Malal	37°23'2.76"S 70°16'23.52″W	2014	MG792044	D	This study
Nqn2_Plottier	Argentina	Neuquén	Plottier	38°57'20.16"S 68°17'22.92″W	2014	MG792046	D	This study
RN_Chimpay	Argentina	Río Negro	Chimpay	39°9'42.84"S 66°7'17.04″W	2014	MG792052	D	This study
Sal1_Cafayate	Argentina	Salta	Cafayate	26°3'47.88"S 65°58'18.84″W	2016	MG792036	D	This study
Sal2_J.V.Gonzalez	Argentina	Salta	J.V.Gonzalez	25°8'54.96"S 64°5'50.64″W	2012	MG792048	D	This study
SFe1_Villa_Eloisa	Argentina	Santa Fé	Villa Eloisa	32°55'35.76"S 61°30'4.32″W	2016	MG792031	D	This study
SFe2_Sunchales	Argentina	Santa Fé	Sunchales	30°58'51.24"S 61°30'39.24″W	2010	MG792039	D	This study
SFe3_Emilia	Argentina	Santa Fé	Emilia	31°3'0.72"S 60°48'47.52″W	2016	MG792053	D	This study
Sgo1_Colonia_Dora	Argentina	Santiago del Estero	Colonia Dora	28°39'3.96"S 63°2'54.24″W	2012	MG792026	D	This study
Sgo2_EEA_Sgo_del_Estero	Argentina	Santiago del Estero	EEA	28°1'20.64"S 64°13'49.80″W	2017	MG792049	D	This study
SJ_Guanacache	Argentina	San Juan	Guanacache	32°2'48.84"S 68°34'4.44″W	2011	MG792028	D	This study
SL1_La_Angelina	Argentina	San Luis	La Angelina	34°17'39.48"S 65°19'32.88″W	2014	MG792021	D	This study

SL2_Lafinur	Argentina	San Luis	Lafinur	32°5'16.44"S 65°26'26.52"W	2014	MG792033	D	This study
Tuc_Tapia	Argentina	Tucumán	Tapia	26°35'9.96"S 65°16'19.56"W	2012	MG792020	D	This study
ENTZ2-3	France	Alsace	Entzeim	48°31'31.43"N 7°38'15.54"E	2017	MH603810	A	This study
VAU14_LUZ142	France	Occitanie	Vauvert	43°34'51.60"N 4°19'5.31"E	2014	KT214373	A	Bernardo et al. 2016
ASS14_Assas2	France	Occitanie	Assas	43°42'6.91"N 3°53'38.37"E	2014	KT214352	A	Bernardo et al. 2016
SSL14_Toul1	France	Occitanie	Saint Sulp. Leze	43°19'45.05"N 1°20'2.12"E	2014	KT214354	A	Bernardo et al. 2016
SSL14_Toul7	France	Occitanie	Saint Sulpice sur Leze	43°19'45.05"N 1°20'2.12"E	2014	KT214357	A	Bernardo et al. 2016
ASS14_Assas1	France	Occitanie	Assas	43°42'6.91"N 3°53'38.37"E	2014	KT214360	B	Bernardo et al. 2016
VAU14_LUZ136	France	Occitanie	Vauvert	43°34'51.60"N 4°19'5.31"E	2014	KT214361	B	Bernardo et al. 2016
MS2-3	France	Occitanie	Rodez	44°20'57.80"N 2°34'33.55"E	2017	MH603811	A	This study
TDV10_44-1E	France	Provence	Sambuc	43°30'3.19"N 4°41'20.78"E	2010	KP732474	A	Bernardo et al. 2016
PB14_GS8	France	Provence	Petit Bastière	43°39'5.61"N 4°34'29.16"E	2014	KT214374	A	Bernardo et al. 2016
TDV14_44-16	France	Provence	Sambuc	43°30'3.19"N 4°41'20.78"E	2014	KT214350	A	Bernardo et al. 2016
PB14_LUZ166	France	Provence	Petit Bastière	43°39'5.61"N 4°34'29.16"E	2014	KT214351	A	Bernardo et al. 2016
BON14_LUZ075	France	Provence	Sambuc	43°30'7.55"N 4°42'45.47"E	2014	KT214353	A	Bernardo et al. 2016
ALB14_LUZ147	France	Provence	Albaron	43°36'20.26"N 4°28'51.49"E	2014	KT214355	A	Bernardo et al. 2016
ALB14_LUZ163	France	Provence	Albaron	43°36'20.26"N 4°28'51.49"E	2014	KT214356	A	Bernardo et al. 2016
PB14_LUZ184	France	Provence	Petit Bastière	43°39'5.61"N 4°34'29.16"E	2014	KT214359	B	Bernardo et al. 2016
GAG14_LUZ193	France	Provence	Gageron	43°35'22.93"N 4°37'55.86"E	2014	KT214362	B	Bernardo et al. 2016
PB14_LUZ182	France	Provence	Petit Bastière	43°39'5.61"N 4°34'29.16"E	2014	KT214363	B	Bernardo et al. 2016
ALB14_LUZ148	France	Provence	Albaron	43°36'20.26"N 4°28'51.49"E	2014	KT214364	B	Bernardo et al. 2016
PB14_GS4	France	Provence	Petit Bastière	43°39'5.61"N 4°34'29.16"E	2014	KT214365	B	Bernardo et al. 2016
ALB14_LUZ164	France	Provence	Albaron	43°36'20.26"N 4°28'51.49"E	2014	KT214366	B	Bernardo et al. 2016
PB14_LUZ171	France	Provence	Petit Bastière	43°39'5.61"N 4°34'29.16"E	2014	KT214367	A	Bernardo et al. 2016
PB14_LUZ188	France	Provence	Petit Bastière	43°39'5.61"N 4°34'29.16"E	2014	KT214368	B	Bernardo et al. 2016
PB14_GS6	France	Provence	Petit Bastière	43°39'5.61"N 4°34'29.16"E	2014	KT214369	B	Bernardo et al. 2016
BON14_LUZ076	France	Provence	Sambuc	43°30'7.55"N 4°42'45.47"E	2014	KT214370	B	Bernardo et al. 2016
PB14_LUZ165	France	Provence	Petit Bastière	43°39'5.61"N 4°34'29.16"E	2014	KT214371	A	Bernardo et al. 2016
PB14_LUZ178	France	Provence	Petit Bastière	43°39'5.61"N 4°34'29.16"E	2014	KT214372	B	Bernardo et al. 2016
PB14_LUZ179	France	Provence	Petit Bastière	43°39'5.61"N 4°34'29.16"E	2014	KT214375	B	Bernardo et al. 2016
TDV12_48-2A	France	Provence	Sambuc	43°30'7.55"N 4°42'45.47"E	2012	KT214358	B	Bernardo et al. 2016
LARAGNE5	France	Provence	Laragne	44°17'52.89"N 5°50'20.03"E	2017	MH603812	B	This study
G10-10_MAP4	Greece	Pella	Apsalos	40°54'07.02"N 22°03'10.06"E	2016	MH603814	A	This study
G8-3_E221	Greece	Thessaloniki	Nymfopetra	40°41'45.08"N 23°20'59.0"E	2017	MH603813	A	This study
G35-4_E215	Greece	Pella	Agios Georgios	40°45'36.0"N 22°15'39.01"E	2017	MH603815	A	This study
MS7-4	Iran	Fars	Shiraz	29°35'30.31"N 52°35'1.67"E	2015	MH603816	C	This study

SH10-4	Iran	Fars	Shiraz	29°35'30.31"N 52°35'1.67"E	2015	MH603817	A	This study
38	Iran	Hormozgan	Bandar Abbas	26°48'54.07"N 55° 6'42.37"E	2015	MH603828	A	This study
6TE-2	Iran	Isfahan	Fooladshahr	32°26'56.75"N 51°24'4.16"E	2016	MH603822	A	This study
ME13	Iran	Isfahan	Filor	32°26'56.75"N 51°24'4.16"E	2015	MH603823	A	This study
ME8	Iran	Isfahan	Filor	32°26'56.75"N 51°24'4.16"E	2015	MH085201	A	Davoodi et al. 2018
12UK	Iran	Kerman	Kerman	29°29'6.03"N 57°38'38.06"E	2016	MH085199	C	Davoodi et al. 2018
1BJ-1	Iran	Kerman	Jiroft	28°39'31.01"N 57°44'3.00"E	2015	MH603824	C	This study
2UK-2	Iran	Kerman	Kerman	29°29'6.03"N 57°38'38.06"E	2016	MH603821	C	This study
51UK	Iran	Kerman	Kerman	29°29'6.03"N 57°38'38.06"E	2016	MH603820	C	This study
C2-2	Iran	Kuzestan	Behbahan	30°35'28.01"N 50°14'5.47"E	2015	MH603818	C	This study
12LO-3	Iran	Lorestan	Noor Abad	34° 3'52.03"N 47°58'32.96"E	2016	MH603819	C	This study
2LO-Q	Iran	Lorestan	Noor Abad	34° 3'52.03"N 47°58'32.96"E	2016	MH603826	A	This study
248-1	Iran	Razavi Khorasan	Sabzevar	36°12'54.64"N 57°40'4.16"E	2017	MH603825	C	This study
254	Iran	Razavi Khorasan	Sabzevar	36°12'54.64"N 57°40'4.16"E	2017	MH603827	C	This study
Kh17-5	Iran	Sistan and Baluchestan	Khash	28°13'53.12"N 61°11'33.94"E	2015	MH603829	C	This study
CO7	Iran	Western Azerbaijan	Urmia	37°40'55.93"N 45°23'39.59"E	2015	MH085200	A	Davoodi et al. 2018
I23-3	Italy	Emilia Romagna	Mirandola	44°52'57.63"N 11° 5'16.32"E	2017	MH603830	A	This study
I25-1	Italy	Emilia Romagna	San Giovanni in Persiceto	44°39'17.42"N 11°10'46.32"E	2017	MH603831	A	This study
I31-2	Italy	Emilia Romagna	Malalbergo	44°42'37.27"N 11°31'16.10"E	2017	MH603832	A	This study
I32-1	Italy	Emilia Romagna	Malalbergo	44°42'37.27"N 11°31'16.10"E	2017	MH603833	A	This study
I42-5J	Italy	Tuscany	Prato	43°54'9.90"N 11° 5'52.42"E	2017	MH603834	A	This study
I44-13	Italy	Liguria	Poggi	44° 4'30.32"N 8°11'12.96"E	2017	MH603835	A	This study
COURM2	Italy	Aosta Valley	Courmayeur	45°46'56.18"N 6°58'14.84"E	2017	MH603836	A	This study
J4-3_JoAl26-17	Jordan	Balqa	Deir Alla	32°11'20.00"N 35°36'11.00"E	2017	MH603837	A	This study
J6-5_JoAl28-17	Jordan	Balqa	Deir Alla	32°11'20.00"N 35°36'11.00"E	2017	MH020805	A	Kumari et al. 2018
J8-1_JoAl30-17	Jordan	Balqa	Deir Alla	32°11'20.00"N 35°36'11.00"E	2017	MH603838	A	This study
J9-1_JoAl31-17	Jordan	Irbid	Ar Ramtha	32°33'39.32"N 36° 0'31.31"E	2017	MH603839	A	This study
L2-6_LAl2_17	Lebanon	Beka'a	Terbol	33°49'3.90"N 35°59'1.38"E	2017	MH603840	A	This study
L3-1_LAl3_17	Lebanon	Beka'a	Terbol	33°49'3.90"N 35°59'1.38"E	2017	MH603841	A	This study
L5-6_LAl22_17	Lebanon	Beka'a	Mansoura	33°40'52.10"N 35°48'56.18"E	2017	MH020806	A	Kumari et al. 2018
ES8-6	Spain	Andalusia	Huétor Tájar	37°11'50.52"N 4° 2'9.96″W	2017	MH603842	A	This study
ES12-8	Spain	Andalusia	Huétor Tájar	37°11'50.28"N 4° 2'10.50″W	2017	MH603843	A	This study
ES17-3	Spain	Andalusia	Huétor Tájar	37°11'51.12"N 4° 1'53.52″W	2017	MH603844	A	This study
ES25-3	Spain	Andalusia	Láchar	37°11'47.70"N 3°51'39.00″W	2017	MH603845	A	This study
ES26-4	Spain	Andalusia	Láchar	37°11'47.76"N 3°51'39.12″W	2017	MH603846	A	This study
ES32-3	Spain	Andalusia	Chauchina	37°11'52.14"N 3°46'52.62″W	2017	MH603847	A	This study

ES34-2	Spain	Andalusia	Chauchina	37°11'52.02"N 3°46'52.56″W	2017	MH603848	B	This study
ES36-5	Spain	Andalusia	Chauchina	37°11'51.96"N 3°46'52.62″W	2017	MH603849	A	This study
ES44-6	Spain	Andalusia	Chauchina	37°11'54.36"N 3°47'20.10″W	2017	MH603850	A	This study
ES52-18	Spain	Andalusia	Chauchina	37°11'52.68"N 3°47'31.26″W	2017	MH603851	B	This study
ES53-1	Spain	Andalusia	Chauchina	37°11'52.68"N 3°47'31.38″W	2017	MH603852	A	This study
S2-9_SyAl35-17	Syria	Hama	Mahrusah	35° 8'37.90"N 36°25'29.66"E	2017	MH603853	A	This study
S4-2_SyAl37-17	Syria	Hama	Mahrusah	35° 8'37.90"N 36°25'29.66"E	2017	MH020803	A	Kumari et al. 2018
S7-1_SyAl40-17	Syria	Hama	Khan Gelmidon	35° 8'37.90"N 36°25'29.66"E	2017	MH603854	A	This study
S12-3_SyAl45-17	Syria	Hama	Jeb Ramleh	35° 3'49.44"N 36°20'30.06"E	2017	MH603855	A	This study
T1-1_TuAl5_17	Tunisia	Ariana	Qal'at Al Andalus	37° 2'10.52"N 10° 7'36.80"E	2017	MH603856	A	This study
T2-1_TuAl6_17	Tunisie	Ariana	Qal'at Al Andalus	37° 2'10.52"N 10° 7'36.80"E	2017	MH020804	A	Kumari et al. 2018
T4-1_TuAl8_17	Tunisia	Ariana	Qal'at Al Andalus	37° 2'10.52"N 10° 7'36.80"E	2017	MH603857	A	This study
T6-1_TuAl10_17	Tunisia	Ariana	Qal'at Al Andalus	37° 2'10.52"N 10° 7'36.80"E	2017	MH603858	A	This study
T12-1_TuAl16_17	Tunisia	Ariana	Qal'at Al Andalus	37° 2'10.52"N 10° 7'36.80"E	2017	MH603859	A	This study
T16-1_TuAl20_17	Tunisia	Béja	Béja	36°43'59.95"N 9°11'3.72"E	2017	MH603860	A	This study
